# Screening for the presence of scleroedema adultorum of Buschke in patients with diabetes mellitus: newly diagnosed patients had a high prevalence of dyslipidaemia

**DOI:** 10.1186/s12944-021-01473-1

**Published:** 2021-05-05

**Authors:** Viktória Csonka, Beáta Bódis, Dániel Kovács, Nelli Farkas, Endre Kálmán, László Czirják, Cecília Varjú

**Affiliations:** 1 Department of Rheumatology and Immunology, Medical School, University of Pécs, Akác u. 1, Pécs, H-7632 Hungary; 2 Department of Internal Medicine, Teaching Hospital Mór Kaposi, Kaposvár, Hungary; 3 1st Department of Internal Medicine, Medical School, University of Pécs, Pécs, Hungary; 4 Department of Vascular Surgery, Medical School, University of Pécs, Pécs, Hungary; 5 Institute of Bioanalysis, Medical School, University of Pécs, Pécs, Hungary; 6 Department of Pathology, Medical School, University of Pécs, Pécs, Hungary

**Keywords:** Scleroedema, Scleroderma-like, Fibromucinous thickening, Diabetes mellitus, Dyslipidaemia, Non-alcoholic fatty liver disease (NAFLD)

## Abstract

**Background:**

Scleroedema adultorum of Buschke is a rare disorder characterized by fibromucinous thickening of the dermis that manifests mainly at the nape of the neck and on the upper back and shoulders. This study screened patients with diabetes mellitus for skin hardening caused by scleroedema adultorum of Buschke and characterized the clinical and laboratory findings in patients with newly identified cases, with a focus on lipid metabolism abnormalities and vascular complications.

**Methods:**

Out of 113 consecutive patients with diabetes, 11 (9.7%) new scleroedema patients, all with type 2 diabetes, were found. Their clinical and laboratory data were compared to those of the rest of the screened patients and to those of a cohort of 15 patients with scleroedema and diabetes who were already being treated in a tertiary clinical centre at the University of Pécs.

**Results:**

Higher proportions of patients with dyslipidaemia, hypertriglyceridemia (*P* < 0.05) and increased mean levels of non-high-density lipoprotein cholesterol (non-HDL-C) were found (*P* < 0.01) in both scleroedema groups than in the group without scleroedema. Stroke and venous thromboembolism (VTE) were more frequently found in the histories of both the newly identified scleroedema group (each 3/11; 27.3%) and the treated cohort (each 6/15; 40.0%) than in the group without scleroedema (6/102; 5.9% in cases of stroke *P* = 0.021, *P* < 0.001; and 14/102; 13.7%; *P* < 0.05 in cases of VTE, respectively). Based on binary logistic regression, a high non-HDL-C level (odds ratio (OD): 3.338, confidence interval (CI): 1.77–6.28; *P* < 0.001) and insulin treatment (OR 7.64, CI 1.9–29.3; *P* = 0.003) were independent predictors of scleroedema in patients with diabetes mellitus.

**Conclusions:**

Diabetes patients with scleroedema had more severe dyslipidaemia and higher occurrence of vascular complications compared to those without scleroedema. In addition to poorly controlled type 2 diabetes mellitus requiring insulin treatment, high non-HDL-C levels may be another contributing factor to the development of scleroedema.

**Trial registration:**

NCT04335396.

## Background

Scleroedema adultorum of Buschke is a scleroderma-like disease characterized by firm, non-pitting oedema that typically begins at the neck and spreads to the shoulders and back [1, 2]. In 1968, Graff described three types of scleroedema [3], although some cases of scleroedema cannot be sorted into these particular categories [4].

The acute form of scleroedema frequently occurs following a febrile illness, usually after respiratory tract infections, and resolves completely within a few months to two years [5, 6]. The second type follows a slow progressive course and is often associated with monoclonal gammopathy or multiple myeloma [7, 8]. Scleroedema type 3 usually develops in patients with poorly controlled, complicated diabetes mellitus and is called scleroedema diabeticorum or scleroedema adultorum of Buschke [9, 10]. The development of scleroedema is usually insidious, and skin thickening does not tend to resolve. Patients are often obese and have one or more complications of diabetes mellitus related to micro- or macro-angiopathy [, 10, 11].

**Fig. 1:**
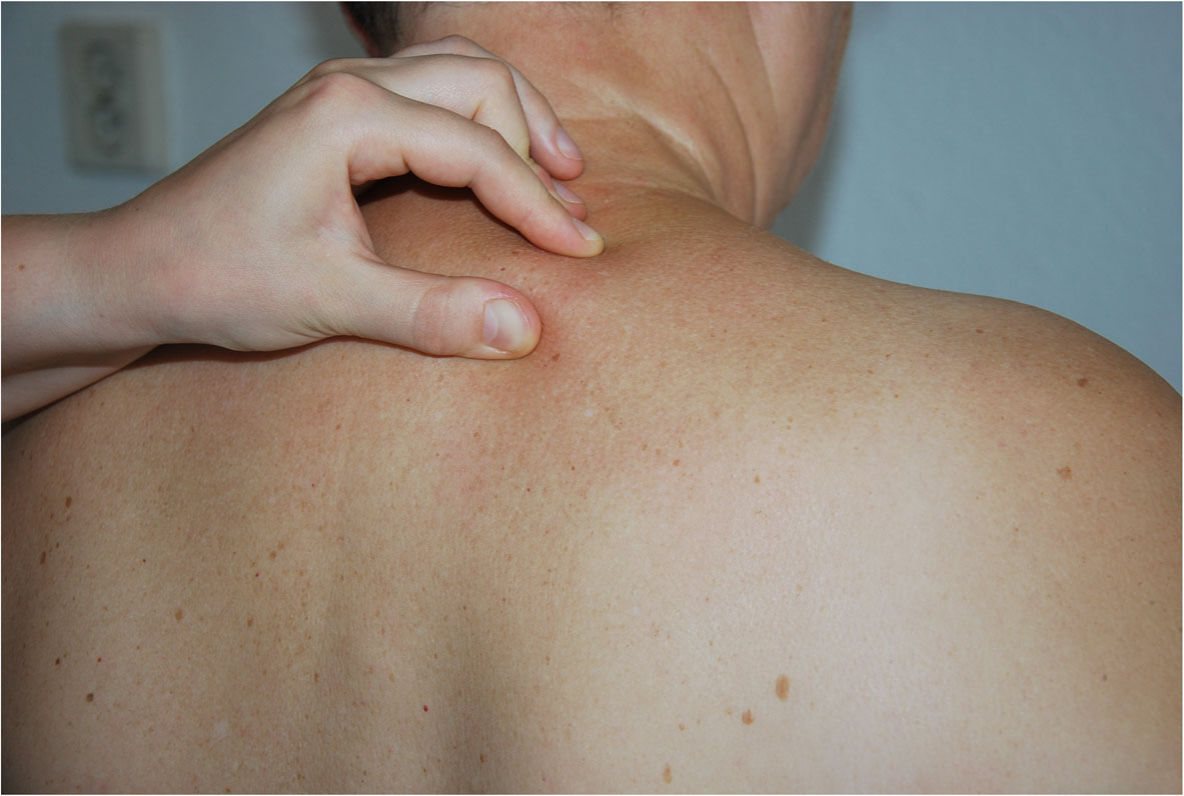
Scleroedema adultorum of Buschke in a patient with diabetes mellitus

**Fig. 2:**
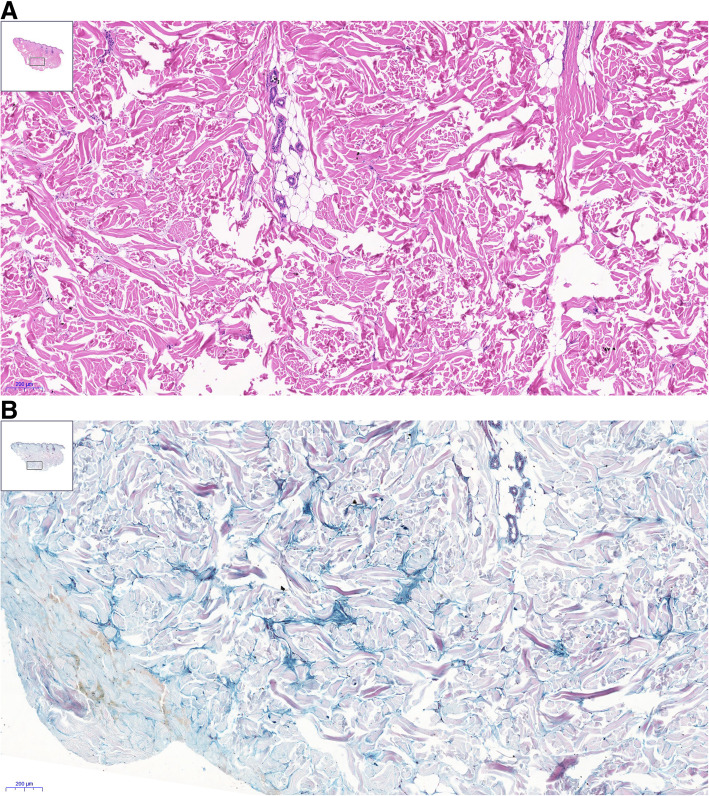
**a** Haematoxylin-eosin staining (5x). Skin sample from the upper part of the back. Deep dermis. The collagen bundles are coarse and separated by apparently empty spaces. Note the overview in the insert in the upper corner. **b** Hale’s colloidal iron staining (5x). Back skin. Deep dermis. The same area as the H&E staining. Between the coarse collagen fibres, the accumulated mucopolysaccharides appear blue. Note the overview in the insert in the upper corner

The affected skin may occasionally become reddish, but it is often only thickened (Fig. 1) [10, 11]. The histopathologic features of scleroedema adultorum of Buschke in patients with diabetes mellitus include thickened collagen bundles within the reticular dermis separated by mucopolysaccharides (mainly mucin) containing fenestrations (Fig. 2a and b) Fibroblasts of the reticular dermis produce an excessive amount of mucin (heavily glycosylated high-molecular weight proteins) and collagens. The overproduction of mucin may be provoked by various factors, including genetic factors, hyperinsulinism and chronic hyperglycaemia. Advanced glycation end-products (AGEs) have been suggested to play a role in scleroedema diabeticorum by causing collagen fibres to become resistant to breakdown [12–15].

Based on two cohorts, the prevalence of scleroedema among patients with diabetes mellitus was 2.5–14.0% [16, 17]. Cole et al. [16] found 12 new patients (2.5%) with scleroedema out of 484 consecutive outpatients with diabetes mellitus. The affected group had a higher proportion of patients with angina pectoris. In their study, 94% of all patients with scleroedema had type 2 diabetes mellitus. In another observational study of 100 hospital-based patients with diabetes mellitus, 14% had scleroedema, including 8 patients with type 1 and 6 with type 2 diabetes, and the group with scleroedema had higher proportions of patients with retinopathy and albuminuria than the group with diabetes mellitus without scleroedema [17]. Patients with scleroedema had a longer duration of diabetes; however, the duration of co-existing scleroedema did not correlate with age or the current mean fasting blood glucose concentration.

In a multicentre study, Rongioletti et al. [18] analysed the medical histories of 44 patients with scleroedema adultorum of Buschke. The majority of them had diabetes mellitus (68%), with a high proportion of patients with poorly controlled type 2 diabetes requiring insulin that was associated with obesity and vascular complications. A relatively high prevalence of thyroid disorders, especially hypothyroidism, was also found in six patients who all had concomitant diabetes.

In this study, patients with diabetes mellitus were screened, and the clinical and laboratory data of the 11 patients with newly identified scleroedema were analysed. Both vascular complications from the case history and the parameters of lipid and carbohydrate metabolism were investigated. Data were also compared between the newly identified patients and another cohort of 15 patients with scleroedema and diabetes who were already being treated in a tertiary clinical centre at the University of Pécs.

## Methods

### Participants

One hundred thirteen Caucasian patients with diabetes mellitus were investigated. The diagnosis of diabetes mellitus was based on the criteria of the World Health Organization (WHO) [19]. The criterion for inclusion was a duration of diabetes mellitus longer than one year. Patients who had taken any form of glucocorticoids in the previous year were excluded. Based on these criteria, 71 females and 42 males, 13 of whom had type 1 and 100 of whom had type 2 diabetes mellitus, were included and screened for scleroedema. Their mean age was 55.9 ± 11.6 years (±S.D.), with a mean duration of diabetes mellitus of 8.1 ± 7.2 years.

Scleroedema was diagnosed by palpation of the skin on the nape of the neck, the back and the shoulders (Fig. 1). The diagnosis of scleroedema was suggested by the physicians performing the screening (CV, VC, DK) and was confirmed by at least one other experienced investigator (CV, LC).

Regarding the 11 patients with newly discovered scleroedema (group S_1_), two were identified from among the 18 consecutive inpatients collected from the 1st Department of Internal Medicine, another two were identified from among the 28 consecutive patients with diabetes mellitus in a local family medicine practice, and seven were identified out of the 67 consecutive patients attending an outpatient clinic for diabetes mellitus. No significant differences were found in the demographic and clinical findings among the patients identified at different sites.

Clinical and laboratory findings of 15 patients with scleroedema diabeticorum who were already being treated in the tertiary care centre were also evaluated (group S_2_) and compared to the newly identified patients. If more than one family member was affected by scleroedema, only the first consecutive patient was enrolled from that particular family. Out of the 15 patients (group S_2_), 6 consented to undergoing a skin biopsy, and all histological analyses were positive for Buschke-type scleroedema.

### Clinical and biochemical data collection

In addition to taking the case history and performing a physical examination, the investigation included measuring the patient’s blood pressure, calculating the body mass index (BMI), and palpating the skin. The patients were also asked to respond to standardized questions about their skin symptoms and previous diabetes-related vascular and neurological complications.

Repeated blood pressure measurements greater than 130/85 mmHg or treatment for previously diagnosed hypertension were categorized as high blood pressure [19]. Polyneuropathy was determined based on an electroneuronography examination in the medical history and/or a physical examination and calibrated tuning-fork test [20, 21]. Significant macro-angiopathy was considered in patients with diagnosed coronary artery disease, cerebrovascular disease, and/or peripheral obliterative angiopathy [22–24]. Significant nephropathy was recorded based on estimated glomerular filtration rate (eGFR) values lower than 60 mL/min/1.73 m^2^ or the presence of micro-albuminuria (> 300 mg/day). Retinopathy was considered based on an ophthalmological examination [14].

Laboratory investigations included the measurement of glycated haemoglobin (HbA1c), parameters of lipid metabolism and thyroid stimulating hormone (TSH) unless the patient already had results for these particular laboratory tests within the past three months.

The most common anti-phospholipid antibodies (aPLs) (anti-cardiolipin IgG, IgM, anti-β2-glycoprotein-I (anti-β2GPI), anti-prothrombin and lupus anticoagulants (LAC) IgG antibodies) were investigated twice in patients with scleroedema.

### Definitions of abnormal lipid metabolism

Atherogenic dyslipidaemia was defined in the case of elevated triglycerides (≥1.7 mmol/L) and decreased HDL cholesterol (< 1.03 mmol/L in males and < 1.29 mmol/L in females) [19, 22].

Non-HDL cholesterol was used as the measurement of atherogenic cholesterol instead of the calculated low-density lipoprotein (LDL) cholesterol to minimize the disruptive effect of higher triglyceride levels. Non-HDL cholesterol was calculated by subtracting HDL cholesterol from the total cholesterol.

Metabolic syndrome was diagnosed based on the International Diabetes Federation Consensus with reference to the global definition of metabolic syndrome [22, 25] based on the following markers: presence of central obesity (waist circumference > 94 cm in males and > 80 cm in females) or a BMI exceeding 30 kg/m^2^ and any two of the following: the presence of elevated triglycerides: ≥1.7 mmol/L; reduced HDL cholesterol (< 1.03 mmol/L in males and < 1.29 mmol/L in females); elevated blood pressure (BP): systolic BP > 130 or diastolic BP > 85 mmHg or treatment for previously diagnosed hypertension; and elevated fasting plasma glucose: > 5.6 mmol/L or previously diagnosed type 2 diabetes mellitus.

The hepatic steatosis index (HSI) was calculated according to Lee et al. [26] as follows: 8 × (alanine aminotransferase/aspartate aminotransferase (ALT/AST) ratio) + BMI (+ 2, if female; + 2, if diabetes mellitus).

The Framingham steatosis index (FrSI) [27] was also calculated as follows:

$$ FSI=-7.981+0.011\times age(years)-0.146\times sex\left( female=1, male=0\right)+0.173\times BMI\left( kg/{m}^2\right)+0.007\times triglycerides\left( mg/ dl\right)+0.593\times hypertension\left( yes=1, no=0\right)+0.789\times diabetes\left( yes=1, no=0\right)+1.1\times ALT/ ASTratio\ge 1.33\left( yes=1, no=0\right). $$

### Statistical analysis

The distribution of variables was evaluated with the Kolmogorov–Smirnov test. Comparisons of clinical data between groups were performed with a Kruskal-Wallis test for non-parametric data or a one-way ANOVA test for normally distributed continuous variables; Bonferroni’s post hoc tests were used in all cases. A chi-square test or Fisher’s test was used for categorical variables. In cases of clinically important outcomes, a power calculation was also performed to estimate the power of the statistical analysis. Clinical and laboratory parameters associated with developing scleroedema were identified with binary logistic regression with stepwise selection. Statistical analyses were performed with the IBM SPSS Statistics v 24.0 software package (IBM Corp., New York, USA).

## Results

### Comparison of clinical and laboratory characteristics in diabetic patients with and without scleroedema adultorum of Buschke

Out of the 113 consecutive patients with diabetes mellitus, 11 patients (9.7%) with scleroedema adultorum of Buschke (group S_1_) were newly identified during screening. The clinical and laboratory data of the scleroedema patients (S_1_) and the rest of the consecutive patients with diabetes mellitus without scleroedema (DM-without-S) are shown in Table 1. Furthermore, the findings in the 15 patients with type 2 diabetes and scleroedema who were already being treated in the centre (group S_2_) are also presented in Table 1.

**Table 1:** Clinical data of patients with diabetes mellitus (DM) with or without scleroedema adultorum of Buschke

	Screened DM cases			***P***-values		
	Patients with newly diagnosed scleroedema (S_**1**_)	DM patients without scleroedema (DM without S)	Scleroedema patients already treated in tertiary care centre (S_**2**_)	S_**1**_ versus (vs.) DM without S	S_**2**_ vs. DM without S	S_**1**_ vs. S_**2**_
Patients, n	11	102	15			
Female, n (%)	8 (73)	63 (64)	7 (46.7)	0.744	0.258	0.246
Age, mean (S.D.) years	63.7 (8.9)	62.3 (12.9)	62.0 (9.6)	0.709	0.942	1.000
Type 2 DM, n (%)	11 (100)	89 (87)	15 (100)	0.358	0.214	N.A.
Type 2 DM duration mean (S.D.), years	20.7 (9.8)	14.2 (11.1)	15.3 (10.2)	0.060	1.000	0.540
BMI median (IQR)	34.2 (11.7)	31.5 (8.7)	34.3 (7.7)	0.050^b^	1.000	0.385
Mean (S.D.)	36.8 (7.3)	31.2 (7.2)	32.5 (5.5)			
Smoking, n (%)	6 (55)	29 (25)	7 (46.7)	0.095	0.229	0.691
Taking insulin, n (%)	8 (73)	37 (39)	7 (46.7)	0.025^d^	0.438	0.246
HbA1c > 6.5%, n (%)	9 (82)	78 (76)	13 (87)	0.634	0.346	1.000
Hypertension, n (%)	10 (91)	82 (80)	14 (93)	0.686	0.301	1.000
Statin users, n (%)	9 (82)	44 (42)	9 (60)	0.024^d^	0.221	0.395
Triglyceride ≥1.7 mmol/L, n (%)	8 (73)	37 (39)	12 (80)	0.025^d^	< 0.001^c^	1.000
Low HDL chol*., n (%)	6 (55)	27 (26)	8 (53.3)	0.328	0.097	1.000
Dyslipidaemia*, n (%)	5 (45)	13 (13)	7 (46.7)	0.015^d^	0.009^d^	0.951
Metabolic syndrome*, n (%)	11 (100)	89 (87)	15 (100)	N.A.	N.A.	N.A.
HSI*, median (IQR)	50.4 (7.6)	42.7 (9.8)	46.8 (4.3)	0.010^a^	0.450	0.529
Mean (S.D.)	50.2 (6.5)	43.1 (8.1)	45.2 (5.5)			
FSI*, median (IQR)	1.12 (1.3)	−0.06 (1.8)	0.83 (0.99)	0.005^a^	0.113	0.852
Mean (S.D.)	1.48 (1.3)	0.06 (1.6)	0.85 (1.06)			
Laboratory findings, means (S.D.)						
HbA1c, %	7.9 (1.7)	8.2 (2.2)	8.6 (1.8)	1.000	1.000	1.000
Triglyceride, mmol/L	2.0 (1.4)	1.7 (1.5)	2.8 (1.2)	0.092	< 0.001^a^	1.000
Cholesterol, mmol/L	5.7 (0.7)	4.7 (1.2)	6.3 (1.2)	0.034^a^	< 0.001^a^	1.000
Non-HDL chol. Mmol/L	4.5 (0.8)	3.4 (1.1)	5.2 (1.5)	0.008^a^	< 0.001^a^	1.000
HDL cholesterol, mmol/L	1.1 (0.3)	1.3 (0.4)	1.1 (0.3)	0.422	0.021	1.000
Uric acid, μmol/L	293 (81)	291 (94)	323 (56)	0.898	0.267	0.846
Creatinine, μmol/L	77.1 (15.4)	78.5 (28.9)	76.0 (19.8)	1.000	1.000	1.000
AST, U/L	24.1 (5.2)	23.0 (15.0)	31.8 (11.8)	0.165	0.002^a^	1.000
ALT, U/L	29.0 (6.1)	25.1 (22.1)	37.3 (15.5)	0.040^a^	< 0.001^a^	1.000
GGT, U/L	39.8 (32.8)	50.8 (64.1)	54.9 (33.8)	1.000	0.120	0.165
ESR, mm/h	25.1 (22.1)	17.9 (15.4)	22.2 (18.2)	1.000	0.582	1.000

*AST* aspartate aminotransferase, *ALT* alanine aminotransferase, *GGT* gamma-glutamyltransferase, *BMI* body mass index, *HSI* hepatic steatosis index [26], *FSI* Framingham steatosis index [27]; *See detailed definitions in Part 2.3 of the Methods; Statistically significant results defined as *P* < 0.05 by ^a^Kruskal-Wallis, ^b^one-way ANOVA, ^c^Chi-square or ^d^Fisher’s exact tests. *N.A*. not applicable, *IQR* interquartile range, *S.D*. standard deviation

**Fig. 3:**
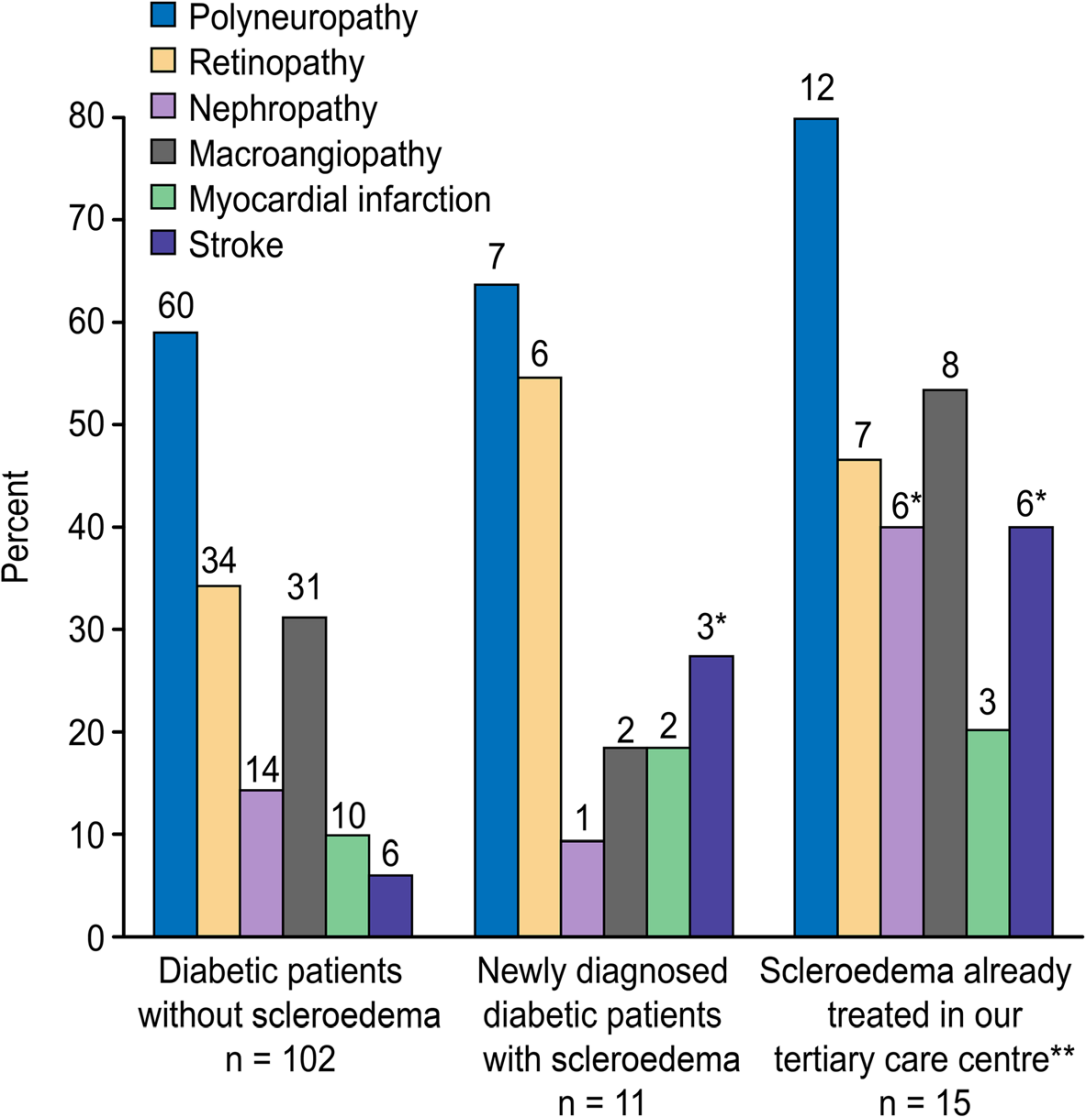
Complications in patients with diabetes mellitus, comparison of patients with and without the presence of scleroedema adultorum of Buschke. *P < 0.05 Scleroedema patients versus diabetes patients without scleroedema. ** Mean follow-up time of the treated patients was 5.5 ± 1.9 years

Examination of the skin involved palpation of the arms, neck and entire trunk. All patients with scleroedema had involvement of the nape and the upper back. Four of the 11 newly diagnosed patients also had thickening of the skin on their shoulders. Skin biopsies were taken for 5 patients, and the results confirmed the presence of scleroedema. None of the newly diagnosed scleroedema patients reported having noticed or complained of skin thickening in their responses to the standardized questions or during the verbal interviews. Generally, the skin affected by scleroedema was not discoloured, but in five patients, it was slightly reddened and became white before returning to red upon palpation. Based on data from the medical histories, group S_1_ had a higher frequency of stroke (*P* = 0.021) than the DM-without-S group; however, no significant differences were observed with regard to myocardial infarction, macroangiopathy, nephropathy, retinopathy and peripheral neuropathy (Fig. 3).

More of the newly identified scleroedema cases had hypertriglyceridemia and dyslipidaemia than those without skin disorders. Patients with scleroedema also had a significantly higher rate of treatment with insulin and statins (Table 1).

Comparison of the S_1_ and DM-without-S groups showed no significant differences in the mean age, sex distribution, prevalence of hypertension, average BMI or mean serum HbA1c level (Table 1). Based on the patients’ medical histories, two patients in the S_1_ group (15.4%) and 11 patients in the DM-without-S group (10.7%) had hypothyroidism; all were treated with thyroid hormone replacement therapy. No significant difference in the number of patients with hypothyroidism was found. The levels of TSH in the sera were within the normal range (0.27–4.2 mIU/L) in both groups.

The mean serum cholesterol, non-HDL-cholesterol and alanine aminotransferase (ALT) levels were significantly higher in the S_1_ group than in the DM-without-S group (Table 1).

The mean serum cholesterol, non-HDL- cholesterol, triglycerides, ALT and aspartate aminotransferase (AST) levels were significantly higher in the S_2_ group than in the DM-without-S group (Table 1). The prevalences of hypertriglyceridemia, dyslipidaemia (Table 1) and previous stroke (*P =* 0.003) were higher in the S_2_ group than in the DM-without-S group (Fig. 3).

In summary, the clinical data in the newly diagnosed (S_1_) and treated (S_2_) groups with scleroedema were similar (Table 1). Patients with scleroedema were more likely than the controls to have hypertriglyceridemia and dyslipidaemia and had higher mean serum levels of non-HDL-cholesterol and ALT. Dyslipidaemia was present in all patients with a history of previous stroke (*n* = 9); these patients belonged to either the S_1_ or S_2_ group (Table 1).

Among the patients with scleroedema, only one had consistently increased anti-prothrombin IgG levels, another patient had a once mildly elevated anti-cardiolipin IgG level, and no patient had increased LAC or anti-β2GPI levels in their sera. The two scleroedema patients with aPLs did not have any thrombotic events in their medical histories. Patients with diabetes without scleroedema were not tested for aPLs in this current study. Venous thromboembolism, represented by deep vein thrombosis and/or pulmonary embolism, occurred more frequently in the scleroedema patients than in the patients without scleroedema, (3/11 (27%) in S_1_, 6/15 (40%) in S_2_ and 14/102 (13.7%) in DM-without-S, *P* < 0.05). Among female patients, there was no significant difference in the occurrence of miscarriage (1/15 (6.7%) vs. 7/65 (10.7%)).

### Comparison of serum lipid parameters in statin users and non-statin users

**Fig. 4:**
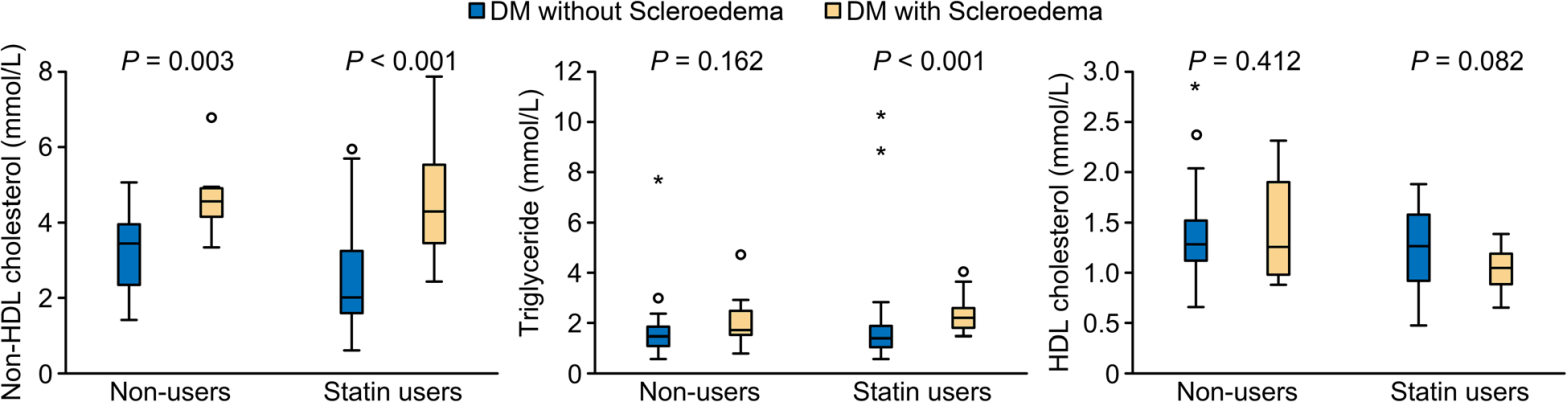
Comparison of serum lipid parameters of patients with diabetes mellitus (DM) with scleroedema (statin users *n* = 18/ non users *n* = 8) and and without scleroedema skin disorders (statin users *n* = 44/ non users *n* = 56). *P*-values were calculated with Mann-Whitney U test; non-HDL-cholesterol: serum non-high-density lipoprotein cholesterol level

Analysis of statin users showed that there were 9 in each of the S_1_ and S_2_ groups; furthermore, there were 44 patients treated with statins in the DM-without-S group. Among statin users, the mean serum levels of non-HDL-cholesterol and triglycerides were significantly higher in both the S_1_ and S_2_ groups than in the DM-without-S group (Fig. 3). When comparing the mean HDL-cholesterol levels, no significant differences were found between the S_1_ and S_2_ groups and the DM-without-S group (Fig. 4). Both the HSI and FSI steatosis indices were higher in the S1 groups compared to patients DM-without-S among the statin users (Table 2).

**Table 2:** Comparison of hepatic steatosis indices between statin users and non-users

	Statin users			Non-users		
	S_**1**_	S_**2**_	DM-without-S	S_**1**_	S_**2**_	DM-without-S
	n = 9	n = 9	n = 44	***n*** = 2	***n*** = 6	n = 56
**Hepatic steatosis index** (IQR)	50.4 (6.5)*	46.8 (5.5)	42.4 (10.1)	51.6 (N.A)	44.6 (9.4)	42.9 (10.6)
**Framingham** **steatosis index** (IQR)	1.12 (0.9)*	0.72 (1.7)	0.024 (1.9)	1.5 (N.A.)	0.15 (2.1)	−0.23 (1.9)

S_1_: newly diagnosed patients with scleroedema out of 113 consecutive patients with diabetes mellitus (DM); S_2_: 15 patients with diabetes and scleroedema adultorum of Buschke treated in a tertiary care centre; DM-without-S: consecutive diabetes patients without scleroedema. *P < 0.05 values are for the comparisons of lipid parameters between patients with scleroedema and those with diabetes without scleroedema. Data are expressed as median (interquartile range, IQR). Mann Whitney U test was applied to calculate *P*-values

Only two patients in the DM-without-S group and none in either of the groups with scleroedema took fibrates.

### Calculation of power for the results

The power ranged between β = 0.62–0.99, where the lowest β value was observed for the comparison of a history of stroke between the S_1_ group and the DM-without-S group, and the highest β values were for the comparisons of the non-HDL cholesterol levels among the three groups.

### Binary logistic regression models identifying the independent predictors of the development of scleroedema in patients with diabetes mellitus

Predictors of scleroedema (groups S1 + S2) were defined by binary logistic regression analysis. First, individual clinical parameters were investigated, and disease duration (OR 1.040, CI 0.98–1.10; *P* = 0.168), serum triglyceride (OR 0.94, CI 0.65–1.35; *P* = 0.733), ALT (OR 1.006, CI 0.98–0.03; *P* = 0.574), BMI (OR 1.087, CI 0.99–1.19; *P* = 0.065), and serum non-HDL cholesterol (OR 2.840, CI 1.68–4.80; *P* < 0.001) were identified as being associated with scleroedema.

Second, predictive factors were assessed using a composite index: HSI, age, disease duration, serum level of non-HDL cholesterol and insulin use (Table 3). In both models, high level of non-HDL cholesterol was associated with scleroedema, and in the second model, insulin use was also associated with the development of scleroedema in patients with diabetes mellitus.

**Table 3:** Clinical and laboratory findings associated with scleroedema

	95% Confidence Interval for Exp(B)			
	Exp (B)	Lower	Upper	***P***
DM duration	1.000	0.937	1.068	0.999
Age	1.011	0.957	1.068	0.694
HSI	1.084	0.996	1.180	0.062
Insulin	7.635	1.987	29.340	0.003
Non-HDL-C	3.338	1.774	6.277	< 0.001

Binary logistic regression analysis with stepwise selection in 26 patients with type 2 diabetes mellitus and scleroedema. *P* < 0.05 represents statistically *significant* values. HSI: hepatic steatosis index, non-HDL-C: serum level of non-HDL cholesterol

## Discussion

### Main findings and interpretation

The prevalence of scleroedema in patients with diabetes mellitus (9.7%) identified in this study was similar to the values reported in previous observational studies (2.5–14%) [16, 17]. In accordance with previous investigations [16–18, 28, 29], high prevalence of cerebrovascular and thrombotic complications were found in patients with scleroedema. None of the patients with newly identified scleroedema had noticed their thickened skin; however, their lipid metabolism parameters were abnormal and were similar to those of the 15 patients who were already being treated in the tertiary care centre. Even in the absence of patient complaints, scleroedema is associated with lipid metabolism disorders (Table 1). Previous investigations showed that patients with scleroedema adultorum of Buschke usually have type 2 diabetes mellitus [17, 18, 28, 29]. The patients with newly identified scleroedema exclusively had type 2 diabetes mellitus.

A lipid profile characteristic on atherogenic dyslipidaemia was found in all groups of patients with diabetes, but those with scleroedema had significantly worse lipid-values (increased mean non-HDL cholesterol and triglyceride, and similar or lower HDL-cholesterol) compared to diabetes patients without scleroedema. The study also showed that all groups had similar HbA1c levels, suggesting that the development of scleroedema may not be exclusively explained by poorly controlled diabetes. Dyslipidaemia may be another factor involved in the development of scleroedema. Koga [30] used a low-density lipoprotein (LDL) apheresis treatment combined with pravastatin and probucol in a patient with severe diabetic scleroedema, hypercholesterolemia and coronary atherosclerotic lesions. After three-years of treatment, the skin involvement was significantly improved, indicating the importance of lipid metabolism in the development of scleroedema.

Several epidemiological studies have shown that cardiovascular diseases, stroke and metabolic syndrome are associated with abnormal levels of liver enzymes, such as ALT and AST [31, 32]. In the liver, the key processes are the overproduction and delayed clearance of triglyceride lipoproteins. Non-alcoholic fatty liver disease (NAFLD) is considered a component of metabolic syndrome, and it is strongly associated with atherosclerosis, cardiovascular disease and stroke [32–34]. The recently developed hepatic steatosis indices (HSI and FrSI) were calculated to detect the presence of NAFLD, and the group with newly diagnosed scleroedema has significantly higher HSI and FrSI scores than the control patients. The treated group with scleroedema (S2) also showed a tendency to have a higher HSI score, although the difference was not significant.

In a previous study [35] thermography was used to detect the circulation of the involved dermal area of a patient with scleroedema. The circulation of the involved skin was improved by administrating daily vasodilator intravenous prostaglandin E1. Based on this observation using vasodilator may be place in the treatment of scleroedema.

A few reports demonstrated more frequent presence of the aPLs in the sera of patients with type II diabetes mellitus. These particular cases had more atherogenic profile with severe micro and/or macrovascular complications, compared to diabetes patients negative for aPLs [36, 37]. In this study aPLs were extremely rare and did not contribute to thrombotic/thromboembolic complications in patients with scleroedema. Since the only significant difference was the higher mean values of BMI of patients with scleroedema (*P* < 0.05), the low-level inflammatory processes associated with obesity might play a role in the increased risk of thromboembolic events.

Using binary logistic regression, a high level of non-HDL cholesterol and insulin therapy were found to be associated with the risk of developing scleroedema in patients with diabetes mellitus; however, it should be mentioned that the associations with HSI and BMI were nearly significant.

Analysis of the statin user subgroup showed that the non-HDL- cholesterol and triglyceride levels were significantly higher in patients with scleroedema than in the control group (Table 2). Statin monotherapy alone did not seem to be effective for the treatment of atherogenic dyslipidaemia in groups with scleroedema; however, it has been clearly described that the adherence to statin treatment is low in the general population [38–41].

### Strengths and limitations of the study

The main strengths of this study are that it is the first systematic evaluation of the connections between scleroedema and lipid metabolism. Independent predictors of the development of scleroedema in diabetic patients were analysed by binary logistic regression. In addition, the recently developed hepatic steatosis indices (HSI and FrSI) were calculated to detect the presence of NAFLD as a component of metabolic syndrome.

However, this study has multiple limitations. First, a relatively small number of patients with skin disorders was included. Second, a low proportion of patients reached the recommended target levels for the lipid parameters. Third, the type and intensity of statin treatment were not analysed in either group.

## Conclusions and clinical relevance

In summary, screening for scleroedema among patients with type 2 diabetes mellitus with a simple physical examination of the skin of the neck and the upper back identified patients who were at higher risk for dyslipidaemia, NAFLD and stroke. Diabetic patients with unnoticed scleroedema were at risk for lipid metabolism disorders. The development of scleroedema is connected with insulin treatment, but it may not be exclusively explained by the poor control of diabetes because haemoglobin A1c levels did not differ between diabetic patients with and without scleroedema.

Scleroedema in diabetic patients could be used to identify individuals prone to cerebrovascular and/or thrombotic complications. Diabetic patients with scleroedema can be classified as being at very high risk for these particular complications. It seems that the standard dosage of statin monotherapy may not be efficient at controlling dyslipidaemia in scleroedema patients. Lifestyle changes, including a proper diet, regular physical activity, and smoking cessation, should be recommended. These very high-risk scleroedema patients need more intensive lipid-lowering therapy with high-intensity statin treatment or combination therapy with ezetimibe or fibrate to achieve the target values for lipid parameters. Anti-platelet therapy can be considered for the prevention of ischaemic stroke, and anti-coagulant treatment can be considered to reduce the risk of recurrent venous thrombotic events in patients with scleroedema. In patients with scleroedema, it is very important to follow the recent recommendations for the treatment of hyperlipidemia [37–41].

## Data Availability

The data set analysed in this study can be reasonably obtained from the corresponding author.

## Abbreviations

Non-HDL-cholesterol: non-high-density lipoprotein cholesterol
NAFLD: Non-alcoholic fatty liver disease
WHO: World Health Organization
AGEs: Advanced glycation end-products
BMI: Body mass index
eGFR: Estimated glomerular filtration rate
HbA1c: Glycated haemoglobin
TSH: Thyroid stimulating hormone
LDL: Low-density lipoprotein
BP: Blood pressure
HSI: Hepatic steatosis index
ALT: Alanine aminotransferase
AST: Aspartate aminotransferase
FrSI: Framingham steatosis index
IgM and IgG: Immunoglobulin G and M
aPLs: Antiphospholipid autoantibodies
anti-β2GPI: anti-β2-glycoprotein-I autoantibody
LAC: Lupus anticoagulants
DM-without-S: Consecutive patients with diabetes mellitus without scleroedema
GGT: Gamma-glutamyltransferase
ESR: Erythrocyte sedimentation rate
OR: Odds ratio
